# Moderate Alcohol Use Is Associated with Reduced Cardiovascular Risk in Middle-Aged Men Independent of Health, Behavior, Psychosocial, and Earlier Life Factors

**DOI:** 10.3390/nu14112183

**Published:** 2022-05-24

**Authors:** Linda K. McEvoy, Jaclyn Bergstrom, Xinming Tu, Alexis C. Garduno, Kevin M. Cummins, Carol E. Franz, Michael J. Lyons, Chandra A. Reynolds, William S. Kremen, Matthew S. Panizzon, Gail A. Laughlin

**Affiliations:** 1 Herbert Wertheim School of Public Health and Human Longevity Science, University of California San Diego, San Diego, CA 92093, USA; jbergstrom@health.ucsd.edu (J.B.); x2tu@health.ucsd.edu (X.T.); agarduno@health.ucsd.edu (A.C.G.); glaughlin@health.ucsd.edu (G.A.L.); 2 Department of Radiology, University of California San Diego, San Diego, CA 92093, USA; 3 Division of Epidemiology and Biostatistics, School of Public Health, San Diego State University, San Diego, CA 92182, USA; 4 Department of Public Health, California State University, Fullerton, CA 92834, USA; kmcummins@fullerton.edu; 5 Department of Psychiatry, University of California San Diego, San Diego, CA 92093, USA; cfranz@health.ucsd.edu (C.E.F.); wkremen@health.ucsd.edu (W.S.K.); mspanizzon@health.ucsd.edu (M.S.P.); 6 Center for Behavior Genetics of Aging, Department of Psychiatry, University of California San Diego, San Diego, CA 92093, USA; 7 Department of Psychological and Brain Sciences, Boston University, Boston, MA 02215, USA; mlyons@bu.edu; 8 Department of Psychology, University of California Riverside, Riverside, CA 92521, USA; chandrar@ucr.edu

**Keywords:** ethanol, CVD, diabetes, metabolic syndrome, atherosclerosis

## Abstract

We examined whether the often-reported protective association of alcohol with cardiovascular disease (CVD) risk could arise from confounding. Our sample comprised 908 men (56–67 years), free of prevalent CVD. Participants were categorized into 6 groups: never drinkers, former drinkers, and very light (1–4 drinks in past 14 days), light (5–14 drinks), moderate (15–28 drinks), and at-risk (>28 drinks) drinkers. Generalized linear mixed effect models examined the associations of alcohol use with three established CVD risk scores: The Framingham Risk Score (FRS); the atherosclerotic CVD (ASCVD) risk score; and the Metabolic Syndrome (MetS) Severity score, adjusting for group differences in demographics, body size, and health-related behaviors. In separate models we additionally adjusted for several groups of potentially explanatory factors including socioeconomic status, social support, physical and mental health status, childhood factors, and prior history of alcohol misuse. Results showed lower CVD risk among light and moderate alcohol drinkers, relative to very light drinkers, for all CVD risk scores, independent of demographics, body size, and health-related behaviors. Alcohol-CVD risk associations were robust to further adjustment for several groups of potential explanatory factors. Study limitations include the all-male sample with limited racial and ethnic diversity, and the inability to adjust for sugar consumption and for patterns of alcohol consumption. Although this observational study does not address causation, results show that middle-aged men who consume alcohol in moderation have lower CVD risk and better cardiometabolic health than men who consume little or no alcohol, independent of a variety of health, behavioral, psychosocial, and earlier life factors.

## 1. Introduction

Despite a large literature spanning decades, the often-reported protective association of alcohol use with cardiovascular health remains hotly debated. Several systematic reviews and meta-analyses of observational studies have shown that light to moderate drinking is associated with reduced risk of cardiovascular disease (CVD) events and mortality, or with lower levels of CVD risk factors [1,2,3,4,5,6]. Short term trials of alcohol use have demonstrated beneficial effects of alcohol on several CVD risk factors, including increasing HDL cholesterol levels, insulin sensitivity, and antioxidant activity, while decreasing fibrinogen levels, platelet aggregation, and inflammatory biomarker levels [7,8,9]. Despite this, the notion that light to moderate alcohol use may have beneficial cardiovascular effects is not universally accepted. Some have argued that beneficial associations in observation studies stem from bias due to use of inappropriate reference groups [10,11] or to inadequate control for confounders [10,12,13]. Clarifying the nature of the association of alcohol use with CVD risk is imperative given the aging of our population, the high and increasing prevalence of alcohol drinking among middle-aged and older adults in the United States [14], and the persistence of CVD as the leading global cause of death and disease burden [15].

One of the main critiques leveled against studies reporting protective associations of alcohol with CVD risk factors or outcomes is the choice of an inappropriate reference group [10,11]. Many studies, particularly older studies, have used an undifferentiated group of current non-drinkers as the reference group. It has long been recognized that current non-drinkers are a heterogeneous group, comprising former drinkers, lifetime abstainers, and very infrequent drinkers. Former drinkers may have quit drinking due to illness or to concerns related to prior problematic drinking [16]. Because former drinkers have been found to have higher risk for CVD mortality than never drinkers [17], inclusion of former drinkers in the reference group can introduce systematic bias towards beneficial associations of alcohol use [4]. Although beneficial health associations of moderate alcohol use have been reported when the reference group was restricted to lifetime abstainers [4,18] these results may also be affected by bias. Lifetime abstinence is rare, and abstainers tend not to be representative of the wider population [19]. Reasons for lifetime abstinence vary and may include factors that confound associations of alcohol use with health outcomes, including poor childhood health, low lifetime socioeconomic status (SES), moral or religious beliefs that may also impact other health behaviors, or having family members with alcohol use disorders, which may increase childhood stress [16,19]. Thus light, infrequent drinkers may be a preferred reference group, as previously suggested [11,20], since they are less likely to systematically differ from other drinking groups, while not drinking sufficiently to experience alcohol’s beneficial biological effects.

In addition to concerns regarding the reference group, there has been concern that observed associations between alcohol and CVD may stem from inadequate control for numerous potential confounders. Individuals who drink in moderation may differ from those who do not drink in numerous ways that may affect cardiovascular health. For example, alcohol use differs by SES, one of the strongest predictors of morbidity and mortality [21]. Moderate drinkers often have higher levels of education and income, and greater access to healthcare than people who do not drink or who drink more heavily. Use of SES proxies such as education level and income may not fully account for differences in financial hardship that impact health outcomes. For example, one study reported that inclusion of direct measures of financial hardship strongly attenuated the beneficial association between moderate alcohol use and self-reported physical health [12].

Differences in health-related behaviors across groups defined by alcohol intake may also bias results. It is well known that those who consume more alcohol are also more likely to smoke, whereas those who abstain from alcohol also tend to abstain from smoking. Moderate drinkers may be more likely to engage in healthy behaviors, such as having a higher quality diet and engaging in regular physical activity. Most studies control for at least some health-related behaviors, particularly smoking, but may omit others, such as diet quality. Moderate drinkers may also enjoy stronger social support, an important contributor to cardiovascular health [22,23]. Moderate drinkers tend to report better physical health, which may be either a cause (i.e., people with poor health may avoid drinking) or a consequence of differences in alcohol consumption. Similarly, differences in levels of depression may be a cause or consequence of alcohol use.

Other potential confounders that impact an individual’s physiological response to alcohol are also important to consider. Biological effects of alcohol differ across individuals according to age, sex, body size, and body composition. Because alcohol is distributed into the total body water compartment, it will be more diluted among individuals with higher body water content (i.e., larger, leaner, younger individuals) [24] and thus associations with quantity of alcohol consumed will vary due to differences in these measures, yet total body water is rarely included as a covariate. Similarly, past history of alcohol consumption may influence associations of current alcohol use and health outcomes, but most studies rely on assessment of alcohol use at a single time point. This can also make it difficult to distinguish former drinkers from lifetime abstainers. Earlier life factors, such as childhood disadvantage, which may impact both later life drinking and later life cardiovascular health, are rarely considered. Similarly, intellectual ability, a strong predictor of cardiovascular mortality [25], is rarely included as a covariate in studies of alcohol and cardiovascular health.

Here we take advantage of the rich variety of measures collected as part of the Vietnam Era Twin Study of Aging (VETSA) to examine associations between alcohol use and CVD risk among a cohort of middle-aged men [26,27] while systematically examining the potential for confounding by a variety of possible explanatory factors. We examined associations of alcohol use with three composite scores that predict risk of future CVD: The Framingham Risk Score (FRS) which was formulated to predict 10-year risk of a variety of CVD events among individuals free of CVD at baseline [28], the atherosclerotic CVD (ASCVD) risk score [29] developed to estimate 10-year risk of a first atherosclerotic CVD event (heart attack or stroke); and the Metabolic Syndrome (MetS) Severity score [30], based on continuous (not dichotomized) values for the five MetS risk factors, which was introduced in 2014 and has been shown to predict long-term risk for CVD, coronary heart disease (CHD), ischemic stroke, and diabetes [31,32,33,34,35].

We tested the hypothesis that, relative to very light drinkers, alcohol intake within current U.S. guidelines for men (up to 2 drinks per day) would show protective associations with the three CVD risk scores and that these associations would be robust to a broad set of potentially explanatory factors including demographic and body size measures, health behaviors, measures of SES, social support, physical and mental health status, childhood factors, and prior history of alcohol misuse. Note that we use the term “protective association” to describe the direction of the hypothesized or observed association of alcohol with CVD risk, not to imply causation.

## 2. Materials and Methods

### 2.1. Study Population

Participants were part of the Vietnam Era Twin Study of Aging (VETSA), a longitudinal study of aging that began collecting data in 2003 [27]. VETSA participants were recruited from the Vietnam Era Twin Registry, a national registry of male twin pairs who served in the U.S. military during the Vietnam war period (1965–1975). VETSA participants are a random sample of the twins who participated in the Harvard Twin Study of Substance Abuse, (the “Harvard Drug Study”; HDS), in 1991–1993 [36]; the HDS did not select on the basis of substance abuse or any other characteristic. VETSA participants are predominantly non-Hispanic white men (91%) who had similar health and lifestyle characteristics at the time of enrollment as other U.S. men in their age range [37]. Although all VETSA participants are veterans, the majority (~80%) did not experience combat [38].

The present study focused on 1261 participants who completed the wave 2 research visit (2009–2013). After excluding four participants without alcohol use data and 174 with incomplete CVD risk factor data, 1083 participants remained. Of these, 175 participants were excluded due to prevalent CVD (defined as self-reported physician diagnosis of heart attack, coronary artery bypass surgery, angioplasty, heart failure, peripheral vascular disease, stroke, or angina), yielding a final analytic sample of 908 men, ages 56–67 years.

### 2.2. Alcohol Use Assessment

Alcohol use was assessed with a structured medical interview. Participants were asked whether they had consumed more than 20 drinks in their life. Those who responded ‘Yes’ were asked to indicate on how many days during the past 2 weeks they drank beer, and on days in which they drank beer, how many bottles of beer they drank. These questions were repeated for wine and hard liquor, asking how many glasses of wine they drank on days they consumed wine, and how many shots of hard liquor they drank on days they consumed hard liquor. The number of drinks of each beverage type was summed to obtain the total number of alcoholic beverages consumed over the past 14 days.

Alcohol use was treated as a categorical variable with six categories defined. *Lifelong abstainers* (“never drinkers”) were defined as those who reported not having consumed more than 20 drinks in their life, who reported no alcohol intake in the current or prior waves, and whose responses on the Diagnostic Interview Schedule (DIS) for the DSM-III-R, administered in 1991–1993 when participants were an average of 44 years old, confirmed minimal or no earlier life alcohol consumption. Former drinkers were defined as those who reported drinking more than 20 drinks in their lives, or who reported drinking alcohol in the prior VETSA wave or in their DIS responses in 1991–1993, but reported no alcohol consumption in the past 14 days. Very light drinkers were defined as those who consumed 1–4 drinks in the past 14 days. This cut-off was chosen to exclude those who may occasionally engage in binge drinking, defined as the consumption of 5 or more drinks in a day. Light drinkers were those who consumed 5–14 drinks, moderate drinkers consumed 15–28 drinks and at-risk drinkers consumed >28 drinks in the past 14 days. Drinkers were classified as having a beverage preference (beer, wine, or hard liquor) if at least 50% of their total consumption was a single beverage type. Those who reported 50-50 consumption of two beverage types were classified as being preferred drinkers for both types.

### 2.3. Assessment of CVD Risk Scores

Three established CVD risk scores were evaluated for the present study: The Framingham Risk Score (FRS) [28], the atherosclerotic CVD (ASCVD) risk score [29], and the Metabolic Syndrome (MetS) Severity score [30]. The factors included in each score and the outcomes they predict are shown in Table 1. Briefly, the FRS is a sex-specific multivariable algorithm for evaluating 10-year risk of developing CVD derived by D’Agostino et al. in 2008 using data from the Framingham Heart Study [28]. FRS defined CVD as a composite of CHD, cerebrovascular events, peripheral artery disease and heart failure. The ASCVD score provides sex- and race-specific 10-year risk estimates of a first hard ASCVD event (fatal or non-fatal heart attack or stroke). It was developed by the ACC-AHA Risk Assessment Council in 2013 using data from several large, racially, and geographically diverse U.S. cohort studies including the Framingham Heart Study, the Atherosclerotic Risk in Communities study, the Cardiovascular Health Study, and the Coronary Artery Risk Development in Young Adults (CARDIA) study [29]. The MetS Severity score (MetS Z-score) is a continuous score derived from the five traditional MetS components using a factor analysis approach and data from adults in the 1999–2010 National Health and Nutrition Examination Survey (NHANES) [30]. For each of the subgroups defined by sex and race/ethnicity, factor loadings from the five MetS components were determined and used to generate equations for computing a standardized MetS severity Z-score. Although the MetS severity score was not formulated to predict risk of a specific outcome, it has been shown to predict long-term risk for CHD, CVD, ischemic stroke, and diabetes beyond that predicted by the individual MetS components [31,32,33,34,35].

In our main analysis, we treated the FRS, ASCVD, and MetS Severity scores as continuous variables to examine differences in the spectrum of CVD risk by alcohol group. In secondary analyses, we analyzed alcohol associations with clinically high CVD risk using specific cut-points to define high CVD risk (i.e., FRS ≥ 20%; ASCVD ≥ 10%). For the MetS severity score, which does not yet have an established clinical cut-off, we selected a threshold of ≥0.5 SD based on prior publications [32,35] and the proportion of participants identified as high risk.

### 2.4. Cardiovascular Risk Factor Assessment for CVD Scores

Height, weight, and waist and hip girths were measured by trained technicians. Systolic and diastolic blood pressures (SBP and DBP) were measured twice in the morning and twice in the afternoon of the same day from seated, rested participants; the mean of the four readings were used in analyses. Antihypertensive medication use was based on self-report and review of medications brought to the clinic visit. Hypertension was defined as SBP ≥ 130, DBP ≥ 85, or use of antihypertensive medication. Fasting plasma glucose, total and HDL cholesterol and triglycerides were measured in certified laboratories (Nichols Institute/Quest Diagnostics, San Juan Capistrano, CA, USA); LDL cholesterol was calculated using the Friedewald equation. Diabetes was defined as fasting glucose > 125 mg/dL, self-report of physician diagnosis or use of diabetes medication. Smoking behavior and race-ethnicity were assessed by standard questionnaires. Black, Hispanic and non-Hispanic white race-ethnicity designations and current smoking (yes/no) were used for deriving CVD and MetS risk scores, as appropriate.

### 2.5. Covariates (Grouped According to Potential Explanatory Categories)

Physical covariates were measured at the study visit by trained technicians using standardized methods. Demographic, psychosocial, behavioral, and health history covariates were assessed through standardized questionnaires and a structured medical history interview administered by the technicians at the study visit. Methods are provided below for base covariates included in all analyses and for each group of potential explanatory factors.

- Base: Age, race/ethnicity, body size/composition, and behaviors.

Race/ethnicity was dichotomized as non-Hispanic white and all others. Body mass index (BMI, kg/m^2^) and waist to hip ratio (WHR) were used as estimates of overall and central adiposity, respectively. Total body water (TBW), a measure of volume of distribution used to determine blood-alcohol concentrations, was calculated using the age-, sex- and ethnicity-specific Watson equation [24,39] and used as an estimate of the influence of age and body size on blood alcohol levels. Health behaviors assessed included smoking, physical activity, and diet quality. Smoking was classified as never, former, or current smoker. Participants were classified as physically active if they reported engaging in moderate to intense physical activity several times per week. Diet quality was assessed as the number of fruits and vegetables consumed each day, categorized as three or more versus fewer.

- Socioeconomic status: Education, cognitive ability, income, financial hardship.

Education was defined as the number of years of formal education completed. Current cognitive ability was defined as the scaled, normalized score on the Armed Forces Qualification Test (AFQT) administered at wave 2 [40,41]. The AFQT is a 100-item general cognitive ability test that is highly correlated (r = 0.84) with standard IQ [42]. Income was based on combined annual family income and included income from wages, retirement and government assistance. It was categorized as <US $40,000, US $40,000–US $90,000, and ≥US $90,000. Participants also reported whether they had “difficulty accessing medical care”, and whether they (and family living with them) experienced “not enough money to meet needs” or “difficulty paying monthly bills” (all yes/no).

- Social Support: Number of confidants, closest friendship quality, marital status/quality.

Aspects of social support were ascertained in separate questions querying the quality of the participant’s relationship with their closest friend and their number of confidants. Based on the distribution of responses, these variables were categorized as rating the closest friendship as having a quality of 8 or more on a 10-point Likert scale (10 being “best possible relationship”), and as reporting 6 or more close confidants, respectively. Marital status/quality was categorized as not married, married with poor perceived quality, or married with good perceived quality.

- Health Status: General health, non-CVD comorbidities, depression, medications.

General health was assessed using the 5-item general health scale from the Medical Outcomes Study 36-item Short Form Health Survey (SF-36) [43]. Total non-CVD comorbidities included self-reported history of physician diagnosis of the following conditions: osteoarthritis, rheumatoid arthritis, peptic ulcer (stomach or duodenal ulcer), pulmonary disease (asthma, chronic bronchitis, or emphysema), liver disease (hepatitis, cirrhosis, or liver damage due to alcohol), kidney disease (eGFR < 60), thyroid disease, and inflammatory bowel disease (Crohn’s disease or ulcerative colitis). Depression was assessed with the Center for Epidemiologic Studies Depression (CES-D) scale, a reliable, 20-item scale to detect symptoms of depression [44]. A CES-D score greater than or equal to 16 indicates risk of clinical depression. Number of medications was the sum of prescribed medications brought to the clinic visit, as well as self-report of any prescribed medications not brought to the clinic visit.

- Childhood factors: Childhood SES, Childhood Disadvantage Index, young adult cognitive ability.

Childhood SES (cSES) was determined based on participant report of parents’ highest levels of occupation and education during their childhood using the Hollingshead-Redlich index [45]. cSES was based on the self-report of both twins, and averaged across all working parents, as previously described [46]. Young adult cognitive ability was defined as the scaled, normalized score on the AFQT administered at the time of military induction (average age of 20 years).

A VETSA childhood disadvantage index (CDI) was developed by Franz and colleagues in 2013 [47] by combining four dichotomous indicators previously used to define childhood hardship [48]: (1) low SES father; (2) low education mother (<12 years of education); (3) large family size (5+ siblings); and (4) family disruption (separation from either or both parents for most of their childhood prior to age 18 due to factors such as parental death, incapacity, separation, or divorce). The number of positive indicators is summed to form a CDI score ranging from 0 to 4.

- Prior history of alcohol misuse: Alcohol dependence, extreme binge drinking.

Information on adverse alcohol use in earlier life was collected as part of the DIS, administered when participants were 44 years old, on average [36]. Diagnosis of alcohol dependence was based on DSM-III-R criteria [49]. Extreme binge drinking was defined as consuming 10 or more drinks in a day.

### 2.6. Statistical Analyses

To characterize the sample, between alcohol-group differences in sociodemographic and health-related measures at study entry were assessed using generalized linear mixed effects models (GLMM) with the identity link for continuous variables, the logit link for binary variables, and the generalized logit link for categorical variables. Family identifier was included as a random effect to adjust for correlated measures among twins.

- Cardiovascular Risk Factor Scores by Alcohol Group.

Associations of alcohol use with CVD risk scores were assessed using GLMM models with the identity link for continuous CVD risk scores and the logit link for dichotomous CVD risk scores, using the very light drinking group as the reference. All models included family identifier as a random effect to account for the correlated nature of measures among twins. Unadjusted analyses included only the CVD risk score under consideration and the family identifier. Base models additionally included demographic measures (age, race/ethnicity), body measures (BMI, WHR, TBW), and health behaviors (diet quality, smoking status, physical activity).

- Testing Potential Explanatory Factors

In separate models, we assessed the influence of each group of potential explanatory factors (SES, social support, health status, childhood factors, and prior history of alcohol misuse) by adding the covariates within each category to the base models. In a final model, we simultaneously adjusted for all potential explanatory factors.

- Sensitivity Analyses.

Whether the alcohol-CVD risk association differed by type of alcohol consumed was examined by running separate base models on the subsets of participants who preferentially consumed beer, wine, or hard liquor, including only those individuals with each beverage preference.

For all analyses, statistical tests were two-sided; *p*-values are reported as continuous measures; multiple comparisons adjustments were not applied. All analyses were run using SAS 9.4 (SAS Institute, Cary, NC, USA).

## 3. Results

### 3.1. Participant Characteristics

Participants were 61.8 (±2.5) years on average (range 56–67 years), with a median family income of $60,000; 60% reported 12 years of education or a high school equivalency diploma. Overall, the majority of participants reported good quality marriages, and a high-quality relationship with their closest friend. Most participants were either overweight (44%) or obese (41%), with a mean BMI of 29.8 kg/m^2^. Overall, 53% had at least one non-CVD comorbidity, the most common were diabetes and osteoarthritis (both 19%). Almost two thirds (64%) of the sample reported consuming alcohol in the past 14 days; only 6% were life-long abstainers (never drinkers), whereas 29% were former drinkers. Among drinkers, beer was the most commonly consumed alcohol beverage (80% of participants), followed by wine (45%), and hard liquor (40%). Although most participants reported consuming more than one type of beverage, 97% had a beverage preference, defined as 50% or greater consumption of one type of alcohol beverage.

Table 2 shows participant characteristics by group. Former drinkers differed from other groups in several attributes: They had the lowest level of education, current family income, cSES, and self-rated health; the highest number of prescription medications; and were least likely to rate their closest friendship as being of high quality. Next to at-risk drinkers, they were most likely to report a history of binge drinking. Never drinkers also differed from other groups on several characteristics: They were least likely to be non-Hispanic White, most likely to be never smokers, to report being in a high-quality marriage, and to report a large number of confidants. Along with very light drinkers, they were least likely to engage in regular physical activity. They also reported the highest self-rated health and used the fewest prescription medications. Very light drinkers did not differ from other current drinking groups except for being less likely to engage in regular physical activity and to have a prior history of alcohol misuse. At-risk drinkers were most likely to be current smokers, least likely to be married, and most likely to have a history of alcohol misuse.

### 3.2. CVD Risk by Alcohol Group

Table 3 shows CVD risk factors by drinking group. HDL cholesterol, SBP, and to a lesser extent, DBP, increased across current drinking groups in parallel with increasing alcohol intake. Total cholesterol levels were highest among at-risk drinkers; triglyceride levels were lowest among light, moderate, and at-risk drinkers. Blood glucose levels and prevalence of diabetes was lowest among light, moderate, and at-risk drinkers. Former drinkers were most likely to have diabetes and hypertension.

Table 4 shows the mean CVD risk scores for each group and the proportions meeting select cut-points for high CVD risk. ASCVD and FRS scores were lowest among both light and moderate drinking groups, whereas MetS Severity scores were lower among light and moderate drinkers and lowest in the at-risk group. Former drinkers had the highest mean scores for each of the three CVD risk scores. Overall, 69% of the sample met criteria for ASCVD risk ≥10%, 44% for Framingham risk ≥20%, and 38% for MetS Severity Z-scores ≥ 0.5 SD. Light, moderate, and at-risk drinkers were the least likely to have either high ASCVD risk or high MetS Severity scores. For the FRS, light and moderate drinkers had the lowest prevalence of high CVD risk individuals.

### 3.3. Association of Alcohol Group with Continuous CVD Risk Scores

**Figure 1:**
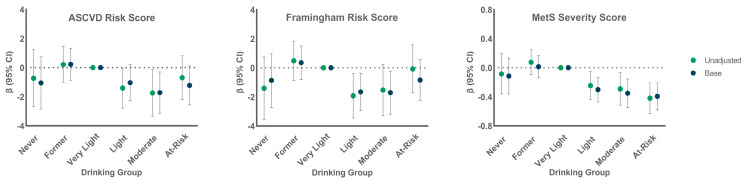
Unadjusted and base model β (95% CI) by drinking group comparing all groups to the very light drinking group for each of the three CVD risk scores.

Figure 1 shows the beta values (95% CI) for each alcohol group relative to the very light drinking group for unadjusted and base models for the three CVD risk scores. The beta values present the mean difference in risk score for each drinking group compared to the very light drinking group; with negative values indicating less risk. For the ASCVD, beta values were below zero for all groups except former drinkers, but only the moderate drinking group had significantly lower ASCVD scores than the very light drinkers. Similarly, FRS scores were below zero for all groups except former drinkers, with significantly reduced risk for light and moderate drinking groups compared to very light drinkers. For the MetS Severity Score, light, moderate, and at-risk drinking groups showed significantly lower scores than the very light drinkers; never and former drinkers did not differ from very light drinkers.

**Figure 2:**
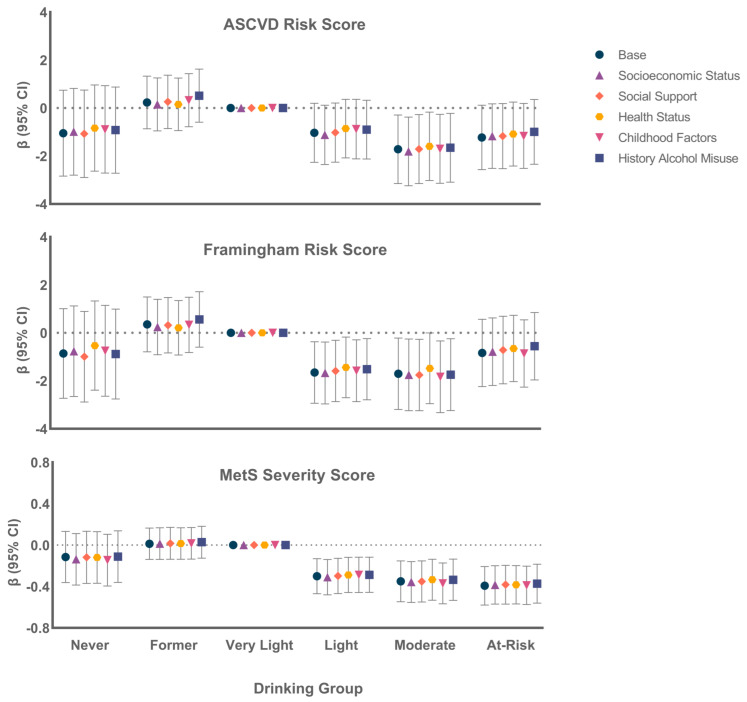
β (95% CI) by drinking group for various explanatory groups, comparing all groups to the very light drinking group, for each of the three CVD risk scores.

Figure 2 shows the results of the different potential explanatory models for each of the risk scores. There was no meaningful change in estimates by adjustment for any group of potential explanatory factors for any score, or for simultaneous adjustment for all explanatory factors (data not shown).

### 3.4. Association of Alcohol Group with High CVD Risk

Table 5 shows the odds ratios (95% CI) for meeting or exceeding specific CVD risk thresholds for each drinking group relative to the very light drinking reference group. Results are shown for unadjusted and base models, followed by additional adjustment for each group of potential explanatory factors. Odds of high ASCVD risk was significantly lower for light and at-risk drinkers relative to very light drinkers in the base models and in all potential explanatory models. Odds of high FRS risk were significantly lower for light and moderate drinkers than very light drinkers in base models and in all explanatory models. The odds of having a high MetS Severity score, was significantly lower for light and moderate groups relative to the very light drinking group in base models and all explanatory models. Although differences were not significant, the likelihood of a high MetS Severity score was greater among never drinkers and former drinkers than very light drinkers in all models.

### 3.5. Association of Alcohol Groups with CVD Risk by Beverage Preference

**Figure 3:**
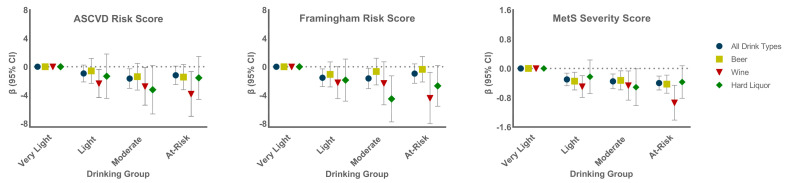
β values (95% CI) by drinking group and drinking type preference, comparing all groups to the very light drinking group, for all three CVD risk scores. Results of the base model are shown.

Overall, 97% of drinkers had a beverage preference (50% or more of one type consumed); 57% preferred beer, 21% wine, and 19% hard liquor; *n* = 32 had a dual 50-50 preference, either beer-wine or beer-hard liquor, and were listed in both categories. Figure 3 shows the CVD risk score beta values (95% CI) for the base model for each drinking group relative to the very light drinking group, first for all drinkers then for drinkers with each beverage preference. There was little difference in ASCVD or FRS scores by drinking group for those who preferred beer. For those who preferred wine, ASCVD and FRS scores were lower among light, moderate, and at-risk groups relative to very light drinkers. Preferential consumption of hard liquor was associated with reduced ASCVD and FRS risk scores among moderate drinkers only. MetS Severity scores were lower among light, moderate, and at-risk drinkers for those who preferred beer, wine and, to a lesser extent, hard liquor.

## 4. Discussion

In this sample of middle-aged men, we found evidence for protective associations of light and moderate alcohol consumption relative to a very light drinking (1–4 drinks in 14 days) reference group with three composite CVD risk scores, the FRS, the ASCVD score, and the MetS Severity score, independent of differences in demographic measures, body size measures, and health-related behaviors. In all cases, associations were not materially changed after further adjustment for a variety of potential explanatory factors, including measures related to SES, social support, physical and mental health status, childhood factors, and earlier life history of alcohol misuse. Exploratory analyses suggested that protective associations were more apparent among wine drinkers than among those who preferred beer or hard liquor for ASCVD and FRS, but that consumption of any type of alcohol was associated with lower MetS Severity scores.

Our purpose in this study was to examine whether the often-reported protective association of light to moderate alcohol intake with cardiovascular health would be apparent when the reference group was restricted to very light drinkers (1–4 drinks in 14 days), and whether any observed associations would be robust to adjustment for a variety of potential confounders. We observed differences in demographic and health characteristics between former drinkers and current drinkers, and between never drinkers and current drinkers, supporting the recommendation to use very light drinkers as the reference group [11,20]. For example, former drinkers experienced poorer health, had the lowest SES (both current and in childhood), and had lower levels of social support than current drinkers, whereas lifetime abstainers were more likely to be non-white, to have never smoked, and to have better social support. In contrast, very light drinkers, who were the largest of the current drinking groups, showed few differences from other current drinking groups, supporting the choice to use this group as the reference to minimize risk of bias.

With the very light drinkers as the reference group, we found protective associations of light, moderate, and in some cases, at-risk drinking, with our three different measures of CVD risk. The FRS and ASCVD include the same seven CVD risk factors (age, sex, SBP, total cholesterol, HDLc, current smoking and diabetes) although they are weighted differently, while the MetS Severity score includes continuous values of the five classic MetS components (SBP, HDLc, triglycerides, glucose, and waist girth). Despite these differences, we found a similar pattern of low FRS, ASCVD, and MetS Severity risk in the light and moderate drinkers, highlighting the robustness of these alcohol associations with lower CVD risk. Protective associations were somewhat stronger for MetS Severity score in continuous analyses, and extended to at-risk drinkers, suggesting that the protective influence of higher alcohol consumption on glucose, HDL cholesterol, and triglycerides outweighed the negative effect on SBP in this sample of middle-aged men.

The magnitude of protective associations observed here, which ranged from 37% to 56% reduction in risk for categorically high ASCVD and FRS, respectively, is somewhat higher, but in the same general range, as pooled estimates from meta-analyses of prospective studies of incident cardiovascular events. For example, in a meta-analysis of 31 studies, Ronksley et al. [3] found a pooled reduction in relative risk of incident CHD of 25%, whereas Roerecke and Rehm [4] reported a pooled reduction in CHD risk of 36% for moderate drinkers relative to lifetime abstainers. Whether inclusion of alcohol use information would improve the predictive ability of CVD risk models may be worthy of examination using prospective data.

The protective associations of light and moderate alcohol intake with the three CVD risk scores were remarkably robust to adjustment for several classes of potential explanatory variables, including SES, social support, health status, childhood factors, and prior history of alcohol misuse. This is consistent with findings from a large meta-analysis that reported that protective associations of moderate alcohol use with incident CHD, and with CVD and CHD mortality, did not differ between studies that adjusted for few potential confounders compared with those that adjusted for many potential confounders [3]. The robustness of our associations to adjustment for known and potential confounders is likely attributable to the fact that light and moderate drinking groups did not differ from the very light drinking reference group on most of these measures. Greater differences in associations between unadjusted and fully adjusted models may be observed when reference groups include former drinkers or are restricted to lifetime abstainers.

Our results show that the association of light and moderate alcohol use with reduced CVD risk is not attributable to confounding by a variety of commonly suggested explanatory measures. There are several plausible biological mechanisms that may underlie the protective associations observed here. As has been reviewed in greater detail elsewhere, alcohol is known to have anti-thrombotic, anti-atherosclerotic, anti-inflammatory, and anti-oxidant effects, as well as to decrease insulin resistance [7,8,50]. There is discrepancy in the literature regarding whether these protective effects arise from alcohol itself, or from other compounds found in alcoholic beverages, such as polyphenols, that differ across beverage types [51,52]. In this respect it is important to note that our sample comprised primarily beer drinkers with relatively few drinkers who preferentially consumed wine or hard liquor. Although we lacked power to determine whether associations differed by beverage type, our exploratory analyses indicated that protective associations were most consistently observed among wine drinkers, in agreement with a Czech study showing better AHA (American Heart Association) Cardiovascular Health scores in individuals who exclusively drank wine [53]. Protective associations among those who preferred beer were only observed for the MetS Severity score, suggesting that compounds other than ethanol may contribute to cardiometabolic aspects of CVD risk protection.

Strengths of our study include the narrow age range of the sample, which minimizes potential differences in associations by age, the availability of prior measures of alcohol use, measurements of multiple CVD risk factors allowing us to assess alcohol associations with three established CVD risk scores, and the ability to evaluate associations while adjusting for a large number of potential explanatory factors, including childhood factors. Weaknesses include the all-male sample with limited racial and ethnic diversity. Our sample was otherwise reasonably representative of American men in this age range during the same time period (2009–2013). For example, median family income was $60,000 in our sample versus $50,022 [54]. While our cohort differed from the average American male at that time in having a higher prevalence of overweight (44% vs. 22%) but less obesity (41% vs. 50%); mean BMI was quite similar (29.8 kg/m^2^ vs. 28.5 kg/m^2^) [55]. Prevalence of alcohol use was 64% versus 68% for U.S. men of similar age [56], and there were fewer abstainers from alcohol use (6% vs. 9.6%) [56]. Although we were able to adjust for a variety of potential confounders, additional confounding from unmeasured, or as yet unidentified factors, cannot be ruled out. For example, we were unable to adjust for differences in sugar consumption, which has a complex association with alcohol use and abstinence [57]. We were also unable to control for differences in pattern of alcohol intake (frequency of drinking, drinking with meals, drinking alone) that may affect associations with health outcomes [58].

In summary, we found that light to moderate alcohol use was associated with reduced 10-year risk of CVD events, and with better cardiometabolic health relative to very light drinking. While this observational study does not address causation, it does clearly show that protective associations of light to moderate drinking can be observed with the use of an unbiased reference group, and that such associations are not due to confounding by a large number of carefully assessed variables that have been previously proposed to account for cardioprotective associations of light to moderate alcohol use.

## Figures and Tables

**Table 1:** Predictors and outcomes predicted for each CVD risk score.

Predictors	ASCVD	Framingham	MetS Severity
Age	X	X	
Sex	X	X	X
Race/ethnicity	X		X
Waist			X
SBP, unspecified			X
SBP, treated	X	X	
SBP, untreated	X	X	
Total cholesterol	X	X	
HDL cholesterol	X	X	X
Triglycerides			X
Current Smoking	X	X	
Fasting glucose			X
Diabetes	X	X	
Outcomes predicted	10-yr risk of nonfatal MI, CHD death, nonfatal or fatal stroke	10-yr risk of nonfatal MI, CHD death, nonfatal or fatal stroke, transient ischemic attack, heart failure, peripheral artery disease	10-yr risk of CVD and CHD

CVD = cardiovascular disease, MetS = Metabolic Syndrome, SBP = systolic blood pressure; CHD = coronary heart disease, MI = myocardial infarction.

**Table 2:** Participant characteristics by drinking group.

	Never (*n* = 57)	Former (*n* = 266)	Very Light (*n* = 197)	Light (*n* = 165)	Moderate (*n* = 100)	At-Risk (*n* = 123)	*p* Value
Demographics							
Age (yrs)	62.5 (2.5)	61.5 (2.5)	61.9 (2.4)	61.5 (2.5)	61.7 (2.5)	62 (2.4)	0.87
Race/ethnicity (% White)	84	88	94	92	93	91	0.01
Body Measures							
BMI (kg/m^2^)	30.2 (4.7)	29.8 (5.5)	29.5 (5)	30.3 (5.2)	30.1 (4.8)	29.1 (4.5)	0.22
Waist girth (cm)	40.8 (4.8)	40.7 (5.3)	40.6 (5.1)	40.9 (4.8)	40.9 (4.6)	40 (4.8)	0.33
WHR	0.97 (0.06)	0.97 (0.06)	0.96 (0.07)	0.96 (0.06)	0.97 (0.06)	0.96 (0.06)	0.27
Total body water (L)	46.2 (5.7)	46.2 (6.4)	46.6 (6.2)	47.2 (6.1)	46.9 (5.4)	45.5 (5.5)	0.22
Health Behaviors							
Smoking (%)							<0.001
Never	87	37	38	35	31	22	
Former	7	46	44	52	52	50	
Current	5	17	18	14	17	28	
Physically active (%)	25	27	25	40	33	36	0.02
3+ servings fruits/veg daily (%)	39	29	38	38	36	28	0.21
Socioeconomic Status							
Education (years)	14.2 (2.3)	13.6 (2.1)	13.9 (2.2)	14.3 (2.1)	13.9 (1.9)	13.8 (2.2)	0.01
Wave 2 AFQT	0.36 (0.71)	0.33 (0.67)	0.38 (0.68)	0.45 (0.66)	0.34 (0.69)	0.26 (0.66)	0.15
Family income (%)							<0.001
<US $40,000	14	27	19	11	15	20	
US $40,000 to $89,999	56	51	50	51	52	49	
≥US $90,000	30	22	30	38	33	32	
Not enough money (%)	11	24	13	14	18	13	0.02
Difficulty paying bills (%)	16	28	17	23	24	20	0.14
Difficulty accessing medical care (%)	2	9	6	7	4	5	0.32
Social Support							
Marital status/quality (%)							0.02
Not married	11	20	16	21	17	28	
Married, poor quality	0	6	9	10	8	7	
Married, good quality	89	74	75	69	75	65	
6+ Confidants (%)	47	30	31	37	25	36	0.07
High quality closest friendship (%)	79	64	79	75	72	70	0.013
Health Status							
SF-36 General Health Score	70.1 (14.9)	63.7 (17.1)	65.6 (16.5)	66.3 (16.1)	69.8 (15.7)	65.7 (14.4)	0.02
Number prescribed meds	2.6 (2.4)	3.8 (3.4)	3.1 (3.1)	2.8 (2.8)	2.8 (3.1)	2.7 (2.3)	<0.01
Number non-CVD comorbidities	0.58 (0.68)	0.88 (0.97)	0.69 (0.86)	0.74 (0.90)	0.72 (0.92)	0.75 (0.82)	0.13
Depression (%)	7	17	14	11	15	15	0.43
Childhood Factors							
Age 20 AFQT	0.34 (0.86)	0.28 (0.69)	0.34 (0.70)	0.43 (0.68)	0.35 (0.69)	0.32 (0.63)	0.31
Childhood SES	32.8 (9.8)	30.8 (10.7)	34.2 (11.7)	32.4 (9.9)	32.9 (10.4)	32.7 (12)	0.04
Childhood Disadvantage							
Low SES father (<33) (%)	56	65	55	60	55	56	0.63
Low education mother (<HS) (%)	45	40	37	32	29	36	0.65
Large family size (5+ siblings) (%)	34	39	35	31	40	38	0.88
Family disruption (%)	7	23	23	18	21	16	0.33
CDI (0–4)	1.37 (1.05)	1.64 (1.13)	1.49 (1.16)	1.39 (1.04)	1.44 (1.18)	1.43 (1.22)	0.59
History of Alcohol Misuse							
DSM-III-R Alcohol dependence (%)	0	34	24	33	42	49	0.001
Extreme binge drinking (10+/dy) (%)	0	27	14	15	20	29	0.001
Current Alcohol Use							
Number drinks past 14 days	-	-	2.4 (1.2)	8.7 (2.9)	21.1 (4.5)	62.1 (39.8)	<0.001
Alcohol type preferred (%)							0.01
Beer	-	-	62	49	63	62	
Wine	-	-	21	31	19	13	
Hard liquor	-	-	17	19	19	26	

Drinking groups are based on number of drinks per 14 days: Very light = 1–4, Light = 5–14, Moderate = 15–28, At-risk = 29+. Values are mean (SD) for continuous variables, proportions for categorical variables. BMI = body mass index, WHR = waist to hip ratio; AFQT = Armed Forces Qualification Test; SES = socioeconomic status, CVD = cardiovascular disease, CDI = Childhood Disadvantage Index.

**Table 3:** CVD risk factors by drinking group.

Risk Factor	Never (*n* = 57)	Former (*n* = 266)	Very Light (*n* = 197)	Light (*n* = 165)	Moderate (*n* = 100)	At-Risk (*n* = 123)	*p*-Value
Waist girth (cm)	40.8 (4.8)	40.7 (5.3)	40.6 (5.2)	40.9 (4.8)	40.9 (4.6)	40.0 (4.8)	0.33
Total cholesterol (mg/dL)	189.6 (45.1)	180 (34.3)	185 (38.3)	182.6 (39.1)	185.9 (35.4)	194.2 (33.2)	0.02
HDL cholesterol (mg/dL)	45.3 (13.8)	44.5 (12.3)	45.4 (11.6)	49.1 (14)	51.5 (15)	58.8 (17.5)	<0.001
LDL cholesterol (mg/dL)	116.3 (40.2)	107.2 (30.3)	111.6 (33.5)	108.9 (32.6)	109.8 (32.4)	108.4 (29.3)	0.29
Triglycerides (mg/dL)	151.2 (154.2)	144.0 (91.0)	144.4 (104.9)	126.1 (88.6)	123.4 (65.3)	136.4 (84.8)	0.08
Glucose (mg/dL)	101.1 (21)	110.9 (38.2)	111.1 (43.1)	103.3 (21.4)	102.9 (23.6)	104.5 (22.4)	0.03
DBP (mmHg)	77.5 (8.5)	78.6 (8.3)	78.2 (7.9)	78.8 (9.7)	79.2 (9)	81.3 (8.4)	0.07
SBP (mmHg)	124.8 (14.1)	127.6 (15.4)	126.8 (13.7)	129.1 (17)	130.1 (15.1)	133.5 (15.9)	<0.01
Anti-hypertensive Medication use (%)	46	54	46	43	53	52	0.22
Hypertension (%)	60	73	70	68	73	80	0.18
Diabetes (%)	17	25	22	15	9	12	0.019

Drinking groups are based on number of drinks per 14 days: Very light = 1–4, Light = 5–14, Moderate = 15–28, At-risk = 29+. Values are mean (SD) for continuous variables, proportions for categorical variables. Risk factors of age and smoking are included in Table 2. DBP = diastolic blood pressure; SBP = systolic blood pressure.

**Table 4:** CVD risk scores and proportions above select risk thresholds by drinking group.

	Never (*n* = 57)	Former (*n* = 266)	Very Light (*n* = 197)	Light (*n* = 165)	Moderate (*n* = 100)	At-Risk (*n* = 123)	*p*-Value
ASCVD Risk Score							
10-year Risk Score	13.5 (5.8)	14.5 (7.3)	14.3 (7.1)	12.9 (6.7)	12.5 (4.8)	13.6 (6.0)	.13
Risk ≥ 10% (%)	68	71	75	59	66	66	0.07
Framingham Risk Score							
10-year Risk Score	18.4 (7.4)	20.3 (7.4)	19.8 (7.2)	17.9 (7.5)	18.3 (6.9)	19.7 (7.5)	0.01
Risk ≥ 20% (%)	40	50	47	35	34	47	0.01
MetS Severity Score							
Z-Score (SDs)	0.34 (0.89)	0.50 (0.93)	0.43 (1.09)	0.18 (0.85)	0.14 (0.82)	0.01 (0.91)	<0.001
Z-Score ≥ 0.5 SD (%)	46	46	39	32	28	29	0.002

Drinking groups are based on number of drinks per 14 days: Very light = 1–4, Light = 5–14, Moderate = 15–28, At-risk = 29+. Values are mean (SD) for continuous variables, proportions for categorical variables. MetS = Metabolic Syndrome.

**Table 5:** Unadjusted and adjusted odds of exceeding specific risk thresholds for each CVD risk score by drinking group. Additional individual models add adjustment for variables grouped by potential explanatory hypotheses.

	Never (*n* = 57)	Former (*n* = 266)	Very Light (*n* = 197)	Light (*n* = 165)	Moderate (*n* = 100)	At-Risk (*n* = 123)
	OR (95% CI)	OR (95% CI)	OR (95% CI)	OR (95% CI)	OR (95% CI)	OR (95% CI)
ASCVD Score ≥ 10%						
Unadjusted	0.71 (0.38–1.36)	0.83 (0.54–1.27)	Ref	**0.48 (0.31–0.75)**	0.64 (0.37–1.10)	0.64 (0.38–1.07)
Base	0.54 (0.25–1.17)	0.83 (0.50–1.37)	Ref	**0.48 (0.29–0.78)**	0.61 (0.31–1.18)	**0.44 (0.25–0.78)**
+ Socioeconomic Status	0.53 (0.24–1.15)	0.76 (0.45–1.26)	Ref	**0.48 (0.29–0.78)**	0.60 (0.30–1.17)	**0.43 (0.24–0.76)**
+ Social Support	0.52 (0.24–1.16)	0.84 (0.50–1.41)	Ref	**0.49 (0.30–0.80)**	0.59 (0.30–1.16)	**0.46 (0.26–0.83)**
+ Health Status	0.57 (0.26–1.25)	0.84 (0.50–1.41)	Ref	**0.51 (0.31–0.83)**	0.63 (0.32–1.22)	**0.45 (0.25–0.79)**
+ Childhood Factors	0.55 (0.25–1.22)	0.81 (0.49–1.35)	Ref	**0.48 (0.29–0.78)**	0.60 (0.30–1.13)	**0.43 (0.24–0.76)**
+ History Alcohol Misuse	0.51 (0.23–1.13)	0.86 (0.52–1.43)	Ref	**0.49 (0.30–0.80)**	0.60 (0.31–1.18)	**0.47 (0.26–0.84)**
FRS ≥ 20%						
Unadjusted	0.74 (0.38–1.45)	1.08 (0.74–1.57)	Ref	**0.59 (0.39–0.90)**	**0.56 (0.34–0.95)**	0.98 (0.62–1.55)
Base	0.76 (0.36–1.53)	1.06 (0.70–1.61)	Ref	**0.54 (0.34–0.86)**	**0.43 (0.22–0.83)**	0.81 (0.48–1.36)
+ Socioeconomic Status	0.79 (0.37–1.66)	1.03 (0.68–1.58)	Ref	**0.54 (0.34–0.85)**	**0.42 (0.21–0.83)**	0.82 (0.49–1.37)
+ Social Support	0.75 (0.34–1.63)	1.03 (0.67–1.58)	Ref	**0.55 (0.34–0.87)**	**0.42 (0.21–0.89)**	0.82 (0.49–1.41)
+ Health Status	0.87 (0.41–1.87)	1.02 (0.67–1.55)	Ref	**0.57 (0.36–0.92)**	**0.44 (0.23–0.87)**	0.86 (0.51–1.46)
+ Childhood Factors	0.80 (0.38–1.71)	1.04 (0.68–1.58)	Ref	**0.55 (0.34–0.87)**	**0.40 (0.21–0.80)**	0.79 (0.47–1.33)
+ History Alcohol Misuse	0.76 (0.35–1.65)	1.12 (0.73–1.73)	Ref	**0.56 (0.35–0.90)**	**0.42 (0.21–0.82)**	0.88 (0.52–1.51)
MetS Severity Score ≥ 0.5 SD						
Unadjusted	1.31 (0.72–2.39)	1.34 (0.92–1.96)	Ref	0.71 (0.46–1.11)	0.60 (0.35–1.04)	0.64 (0.39–1.06)
Base	1.26 (0.67–2.40)	1.28 (0.82–2.00)	Ref	**0.57 (0.34–0.95)**	**0.46 (0.24–0.88)**	0.64 (0.36–1.14)
+ Socioeconomic Status	1.32 (0.67–2.60)	1.31 (0.84–2.05)	Ref	**0.57 (0.34–0.95)**	**0.44 (0.23–0.86)**	0.60 (0.34–1.08)
+ Social Support	1.33 (0.68–2.60)	1.31 (0.83–2.08)	Ref	**0.57 (0.34–0.97)**	**0.45 (0.23–0.88)**	0.64 (0.36–1.13)
+ Health Status	1.33 (0.68–2.59)	1.32 (0.84–2.07)	Ref	**0.59 (0.35–0.99)**	**0.47 (0.24–0.92)**	0.61 (0.34–1.10)
+ Childhood Factors	1.31 (0.66–2.60)	1.36 (0.86–2.14)	Ref	0.62 (0.37–1.04)	**0.44 (0.22–0.86)**	0.62 (0.35–1.12)
+ History Alcohol Misuse	1.34 (0.68–2.64)	1.36 (0.86–2.13)	Ref	**0.59 (0.35–0.99)**	**0.48 (0.24–0.94)**	0.64 (0.36–1.14)

Drinking groups are based on number of drinks per 14 days: Very light = 1–4, Light = 5–14, Moderate = 15–28, At-risk = 29+. Bolded values indicate statistically significant associations.

## Data Availability

Data from VETSA wave 2 are available from the Vietnam Era Twin Study of Aging (see website for data requests: https://medschool.ucsd.edu/som/psychiatry/research/VETSA/Researchers/Pages/default.aspx, accessed on 19 Feburary 2021). Data from earlier life are available with permission from the Vietnam Era Twin (VET) Registry (see https://medschool.ucsd.edu/som/psychiatry/research/VETSA/Researchers/Pages/default.aspxand, accessed on 19 Feburary 2021; https://www.seattle.eric.research.va.gov/VETR/Home.asp, accessed on 19 Feburary 2021).
